# The Chemokine MIP1α/CCL3 Determines Pathology in Primary RSV Infection by Regulating the Balance of T Cell Populations in the Murine Lung

**DOI:** 10.1371/journal.pone.0009381

**Published:** 2010-02-24

**Authors:** John S. Tregoning, Philippa K. Pribul, Alasdair M. J. Pennycook, Tracy Hussell, Belinda Wang, Nicholas Lukacs, Jurgen Schwarze, Fiona J. Culley, Peter J. M. Openshaw

**Affiliations:** 1 Department of Respiratory Medicine, The Centre for Respiratory Infections Research and the MRC & Asthma UK Centre in Allergic Mechanisms of Asthma, National Heart and Lung Institute, Imperial College London, London, United Kingdom; 2 Department of Pathology, University of Michigan Medical School, Ann Arbor, Michigan, United States of America; 3 Centre for Inflammation Research, University of Edinburgh, The Queen's Medical Research Institute, Edinburgh, United Kingdom; Ludwig-Maximilians-Universität München, Germany

## Abstract

**Background:**

CD8 T cells assist in the clearance of respiratory syncytial virus (RSV) infection from the lungs. However, disease after RSV infection is in part caused by excessive T cell activity, and a balance is therefore needed between beneficial and harmful cellular immune responses. The chemokine CCL3 (MIP1α) is produced following RSV infection and is broadly chemotactic for both T cells and natural killer (NK) cells. We therefore investigated its role in RSV disease.

**Methodology/Principal Findings:**

CCL3 was produced biphasically, in both the early (day 1) and late (day 6–7) stages of infection. CCL3 depletion did not alter the recruitment of natural killer (NK) cells to the lungs during the early stage, but depletion did affect the later adaptive phase. While fewer T cells were recruited to the lungs of either CCL3 knockout or anti-CCL3 treated RSV infected mice, more RSV-specific pro-inflammatory T cells were recruited to the lung when CCL3 responses were impaired. This increase in RSV-specific pro-inflammatory T cells was accompanied by increased weight loss and illness after RSV infection.

**Conclusions/Significance:**

CCL3 regulates the balance of T cell populations in the lung and can alter the outcome of RSV infection. Understanding the role of inflammatory mediators in the recruitment of pathogenic T cells to the lungs may lead to novel methods to control RSV disease.

## Introduction

Respiratory Syncytial Virus (RSV) is the leading cause of infant hospitalization [1], [2]. Currently, there is no vaccine against RSV and the only specific intervention for RSV is a virus-specific monoclonal antibody. The bronchiolitis and airway occlusion that can result from RSV infection are believed to be immunopathological in nature, because large numbers of inflammatory cells are recruited to and activated in the lungs [3], [4]. The contribution of the immune system to the bronchiolitis seen during RSV infection opens up possible therapeutic options based on dampening the pathogenic immune response. T cells have been demonstrated to be an important part of this pathogenic inflammatory infiltrate [5]; therefore, inflammatory mediators which recruit T cells to the lung are candidates for novel therapeutics. However, T cells recruited following RSV infection can be either pro-inflammatory [6] or regulatory [7], [8] with the consequence that interventions that lead to reduced recruitment of regulatory T cells may increase inflammation.

One potential target for intervention is CCL3 (MIP1α), chemotactic for both T cells and natural killer (NK) cells. *In vitro*, CCL3 is produced following RSV infection of airway epithelial cells [9]. CCL3 mRNA and protein are both detected in bronchoalveolar lavage (BAL) samples of infants infected with RSV [10], and CCL3 protein levels correlate with the severity of hypoxia in bronchiolitis [11]. We have previously shown that CCL3 is significantly upregulated both during primary murine RSV infection [12], [13] and in enhanced immunopathology after antigenic sensitization [14].

In the current study we observed a biphasic release of CCL3 during primary RSV infection. The early production is from macrophages and the later phase from CD8 T cells. Depletion of CCL3 (by antibody or CCL3 knockout) during RSV infection reduced T cell recruitment, did not affect NK cell responses but increased recruitment of RSV-specific CD8 cells making TNF and IFNγ, thereby increasing disease severity.

## Results

### The Production of CCL3 Following RSV Infection Is Biphasic

**Figure 1:**
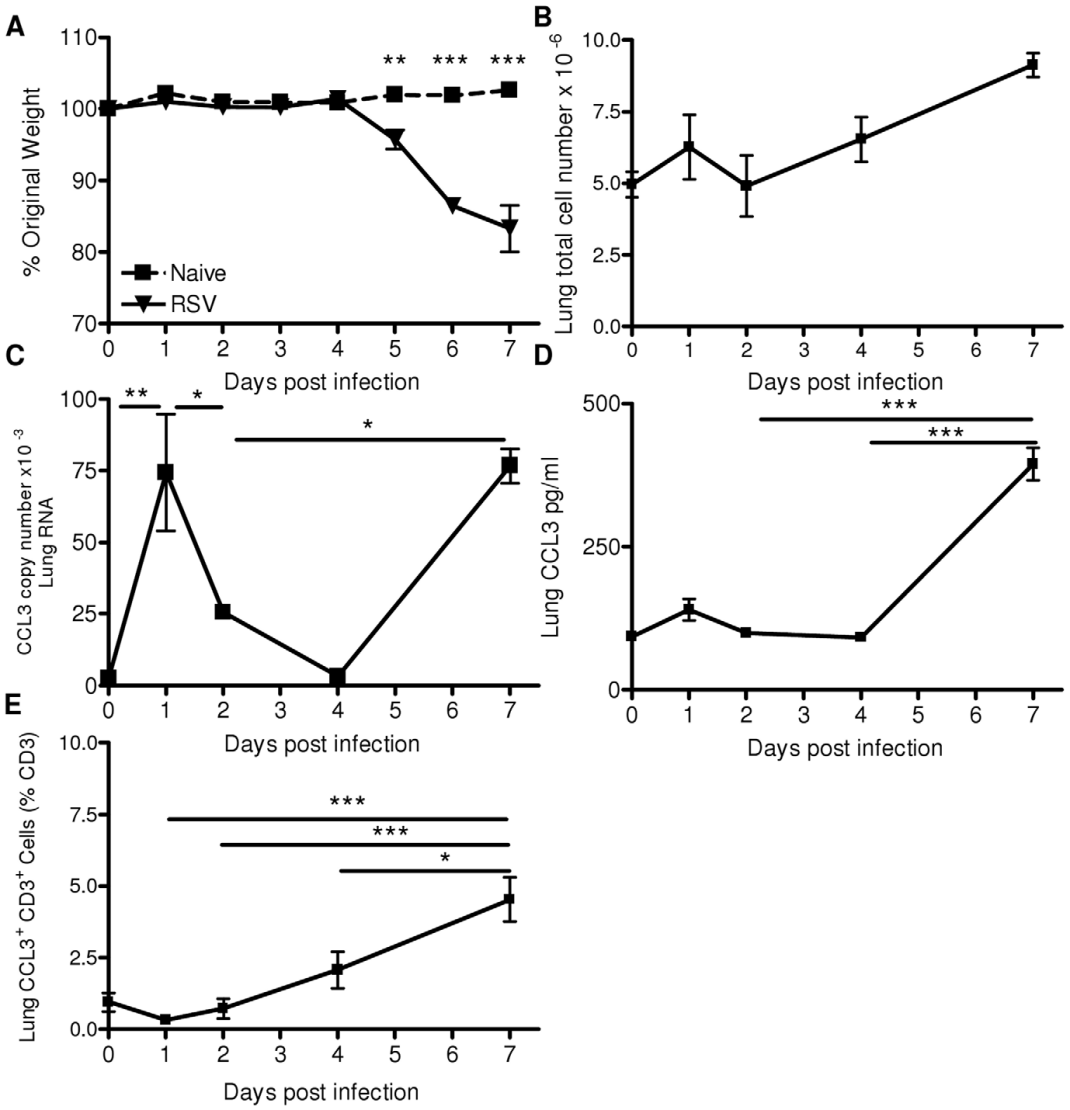
CCL3 is produced biphasically following RSV infection. Mice were infected intranasally (i.n.) with 5×10^5^ PFU RSV. Weight change after RSV infection (A). Time course of cell recruitment (B), CCL3 RNA (C) protein (D) in the lung and RSV M2 (_82-90_) peptide stimulated CD3 T cells stained for intracellular CCL3 (E). Points represent mean of n≥4 mice ± SEM, p<0.05, ** p<0.01, *** p<0.001.

As observed previously [13], RSV infection led to significant weight loss on days 6 and 7 post infection (p.i.; Figure 1a), when pulmonary cell recruitment also peaked (Figure 1b). However, CCL3 RNA in lung tissue showed a double peak on days 1 and 7 p.i. (Figure 1c). CCL3 protein concentration was measured in the sera, nasal wash, BAL and lung homogenate supernatant. None was detected in sera or nasal wash, but in the BAL there was a peak of CCL3 at day 0 and a second peak on day 7 (not depicted). In lung homogenate there were also two peaks of CCL3 production on days 1 and 7 p.i. (Figure 1d). Using intracellular staining to detect CCL3 in CD3^+^ T cells, following RSV peptide stimulation, a peak of CCL3 production was seen on day 7 p.i. (Figure 1e).

### CCL3 Does Not Contribute to Innate Effector Cell Recruitment Following RSV Infection

**Figure 2:**
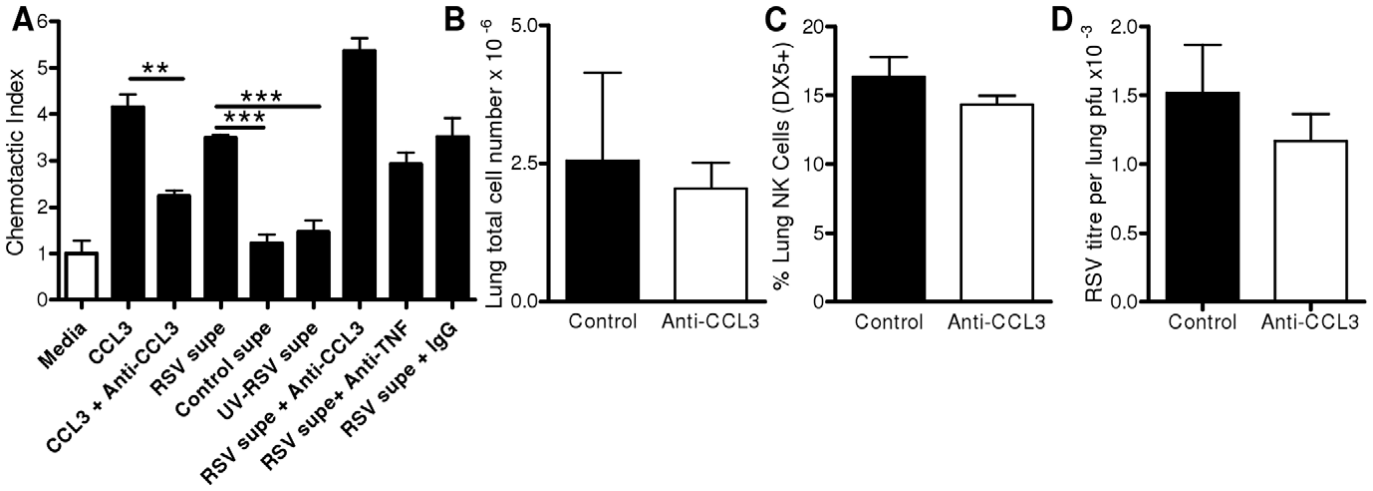
CCL3 is not involved in the recruitment of NK cells during RSV infection. Chemotaxis of enriched NK cells to recombinant CCL3 or supernatant (supe) from RSV, UV-RSV, or control exposed macrophages; anti-CCL3, anti-TNF or control antibody was used to assess the chemotactic effect of specific mediators (A). BALB/c mice were treated on day −1 and +1 of RSV infection with anti-CCL3 (white bars) or control Ig (black bars). Lung cell number (B), percentage lung NK cells (C) and lung viral load (D) on day 4 post infection. Bars represent n≥3 ± SEM, ** p<0.01.

In previous studies we described an early peak of inflammatory mediator production from alveolar macrophages, which was important in the recruitment of NK cells [13]. To determine the contribution of CCL3 to NK cell recruitment, we used a chemotaxis assay. NK cells were enriched using negative magnetic cell sorting from spleen lymphocytes prepared by Ficoll-Paque separation. The enriched cells were added to chemotaxis assay plates to which the potentially chemotactic medium had been added. After incubation, cell migration was quantified by flow cytometry. We found that NK cells were recruited by CCL3 (Figure 2a) and CCL2 (data not depicted). There was no increase in NK cell detection when cells were pre-mixed with CCL3, indicating an increase in chemotaxis rather than chemokinesis (data not depicted).

We saw no chemotaxis towards bronchoalveolar lavage (BAL) collected from RSV infected mice at 24 hours post RSV infection (data not depicted). However the levels of CCL3 measured in the BAL were at 4 pg/ml and the lowest level we used in the chemotaxis assay was 62.5 ng/ml. As BAL supernatant provided too low a concentration of chemokines for NK cell chemotaxis, we used the supernatant from enriched alveolar macrophages that had been exposed to RSV. This supernatant caused chemotaxis equivalent to that of 200 ng/ml CCL3 and significantly greater than chemotaxis caused by supernatant from control alveolar macrophages (p<0.001, Figure 2a). The supernatant from alveolar macrophages incubated with UV inactivated RSV had no effect on chemotaxis. Treatment of supernatant with either anti-CCL3 or anti-TNF did not significantly affect NK chemotaxis.

The same effect was observed using *in vivo* studies. Treatment of mice with anti-CCL3 prior to RSV infection did not significantly alter cell recruitment on day 4 post infection (Figure 2b) nor the percentage of NK cells recruited (Figure 2c). No difference was seen in the peak viral load by plaque assay on day 4 following anti-CCL3 treatment (Figure 2d) or in CCL3^−/−^ knockout mice compared to wild type (data not depicted), this was confirmed by RSV specific qPCR estimation of viral RNA levels in lung homogenate.

### CCL3 Is Important in the Recruitment of T Cells to the Lung

**Figure 3:**
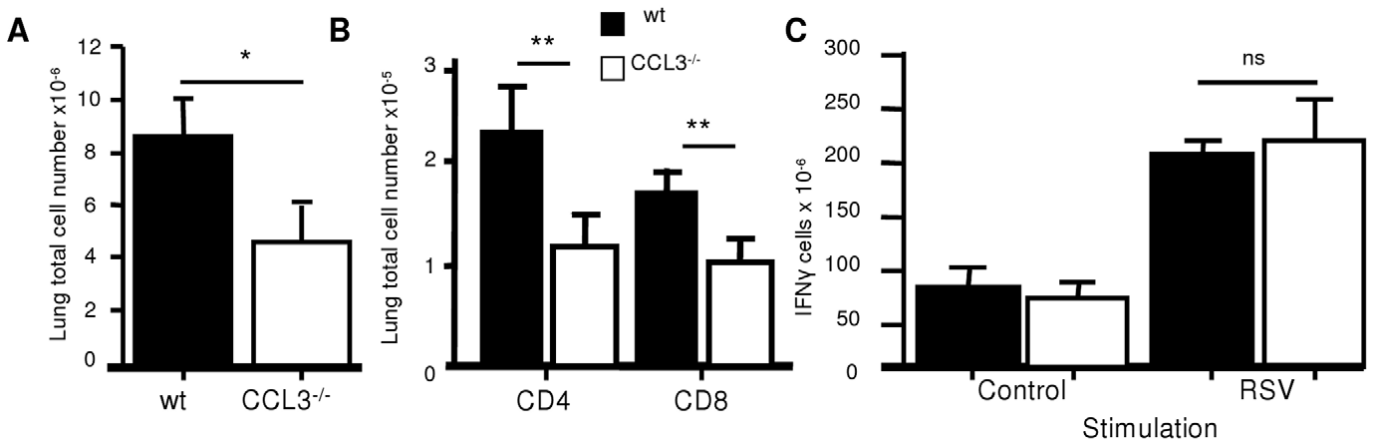
CCL3^−/−^ knockout mice have reduced total cellular recruitment without altering RSV specific cell number. CCL3^−/−^ (white bars) or wild type C57BL/6 control (black bars) mice were infected i.n. with RSV. Lung cell number (A) and percentage of lung CD4 and CD8 + cells on day 7 p.i. (B). RSV specific IFNγ secretion measured by lung cell ELISPOT at day 7 p.i. (C). Points represent n≥4 mice ± SEM, * p<0.05, ** p<0.01.

C57BL/6 mice genetically deficient in CCL3 (CCL3^−/−^), were used to analyze the response to RSV in the absence of CCL3. There was no detectable CCL3 mRNA in CCL3^−/−^ mice (data not depicted). During primary infection CCL3^−/−^ mice showed significantly reduced cell recruitment to the lung (p<0.05, Figure 3a) compared to wildtype C57BL/6 mice on day 7. The recruitment of CD4 and CD8 T cells in the lung was significantly reduced in CCL3^−/−^ mice (p<0.01, Figure 3b). There was no difference in the number of RSV specific IFNγ secreting cells measured by ELISPOT (Figure 3c).

**Figure 4:**
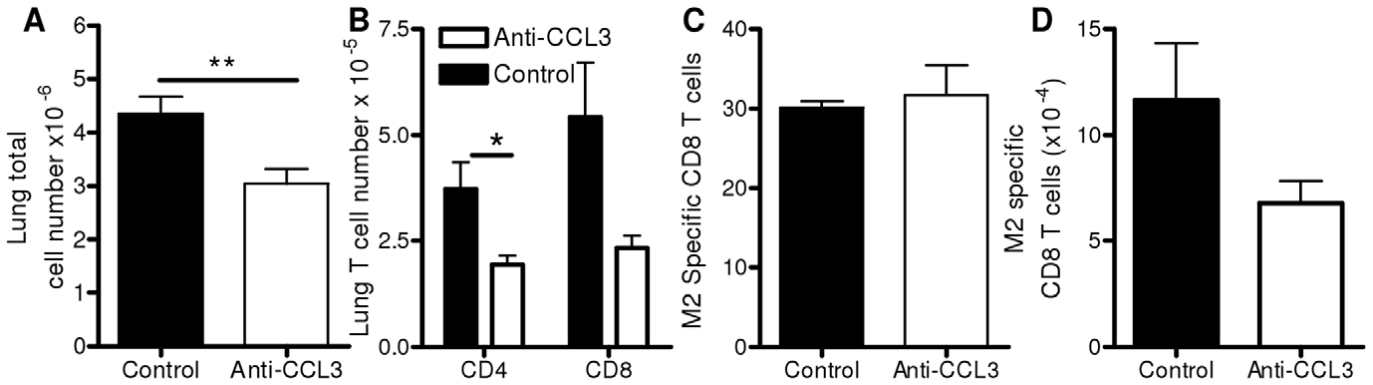
CCL3 depletion reduces cell recruitment without changing RSV specific cell number. BALB/c mice were treated on day −1 and +1 of RSV infection with anti-CCL3 (white bars) or control Ig (black bars). Lung cell numbers (A) and percentage of lung CD4 and CD8+ T cells on day 7 p.i. (B). Proportion (C) and total number (D) of RSV specific T cells in lung measured using RSV (M2) specific pentamer. Points represent n≥4 mice ± SEM, * p<0.05, ** p<0.01.

Since BALB/c mice respond to RSV infection with more pronounced pathology than C57BL/6 and have well characterized CD8 epitopes, we used CCL3 depletion by antibody in this strain to assess the role of CCL3 in RSV infected BALB/c mice. Mice treated with anti-CCL3 on day −1 and +1 of RSV infection showed reduced cellular recruitment to the lungs on day 7 p.i. (p<0.01, Figure 4a), due to reduced numbers of both CD4 (p<0.05) and CD8 T cells (Figure 4b). As in CCL3^−/−^ mice, there was no change in the proportion of RSV specific T cells as shown by detection of RSV M2 peptide (M2_82−90_) specific cells (Figure 4c). However, the total number of M2 specific CD8 cells in the lungs was reduced in anti-CCL3 treated mice (Figure 4d) reflecting reduced cell numbers.

**Figure 5:**
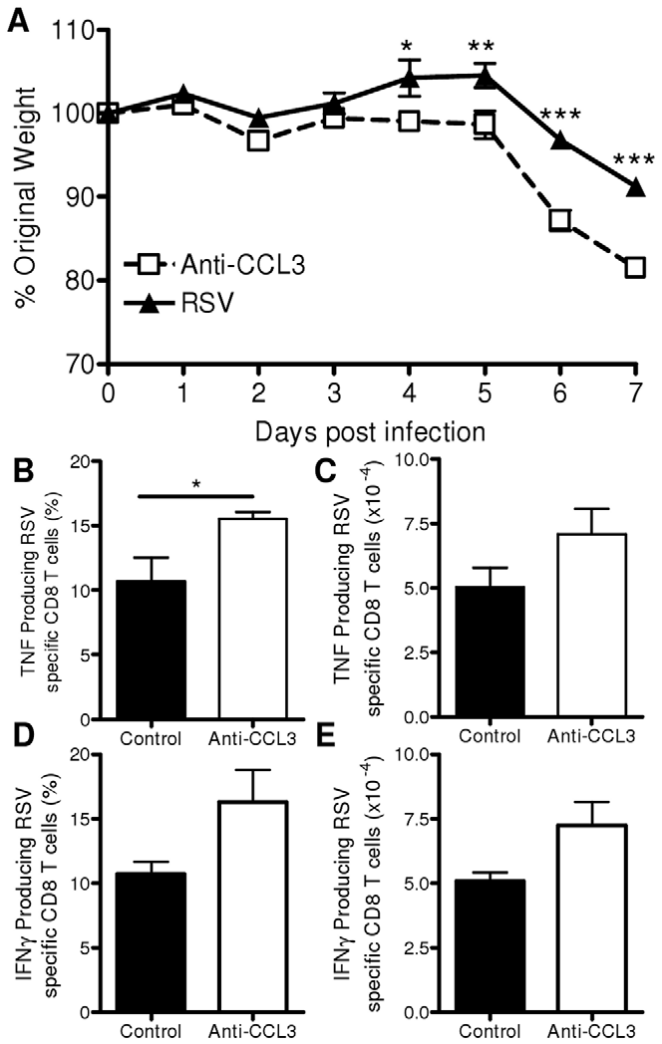
CCL3 depletion increases pathology following RSV infection. BALB/c mice were treated on day −1 and +1 of RSV infection with anti-CCL3 (white bars/squares) or control Ig (black bars/triangles). Weight change after RSV infection (A). Lung cells were stimulated with the RSV M2 (_82-90_) peptide to determine RSV specific responses on day 7 p.i.; percentage (B) and total (C) TNF producing CD8 cells, percentage (D) and total (E) IFNγ producing CD8 cells. Points represent n≥4 mice ± SEM, * p<0.05, ** p<0.01, *** p<0.001.

RSV-infected mice treated with anti-CCL3 lost significantly more weight than control mice on days 6 and 7 p.i. (p<0.001, Figure 5a). RSV specific production of cytokines by CD8 T cells was measured following stimulation with the immunodominant RSV M2 peptide. At the peak of weight loss (day 7) there was a significantly greater proportion (p<0.05, Figure 5b) and number (Figure 5c) of RSV specific TNF producing CD8 T cells after CCL3 depletion. There was also an increased percentage (Figure 5d) and total number (Figure 5e) of RSV specific IFNγ producing CD8 cells.

### TNF Production Contributes to RSV Pathology

**Figure 6:**
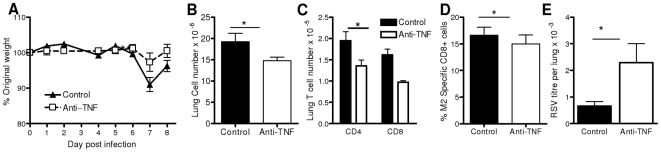
TNF depletion decreases pathology following RSV infection. Weight change after RSV infection (A). BAL cell number (B). Percentage of lung CD4^+^ and CD8^+^ cells (C), RSV M2 pentamer specific lung CD8 T cells (D), measured on day 7 p.i. using flow cytometry. Lung RSV titer on day 4 p.i. measured by plaque assay (E). Points represent n≥4 mice ± SEM, * p<0.05, ** p<0.01, *** p<0.001.

Since TNF producing CD8 cells increased in parallel with the increased weight loss, we looked at whether TNF depletion inhibited RSV pathology. Mice treated with anti-TNF lost significantly less weight than control mice at the peak of disease (p<0.01, Figure 6a). There was also significantly reduced total cell recruitment to the airways (p<0.05, Figure 6b). CD4 and CD8 T cell recruitment to the airways was also significantly reduced (p<0.001, Figure 6c). Anti-TNF treatment reduced the number of RSV specific CD8 T cells recruited to the lungs (p<0.05, Figure 6d) and increased viral load – measured by plaque assay (p<0.05, Figure 6e).

## Discussion

Here we demonstrate that CCL3 was produced biphasically during RSV infection. It is known that other chemokines are also produced in a biphasic manner [12], [14] and that the early peak of chemokine is principally macrophage derived [13]. The early wave of mediator release causes innate effector cell recruitment particularly neutrophils and NK cells. However, both *in vitro* and *in vivo* we saw no contribution of CCL3 to the recruitment of NK cells suggesting that other mediators produced by macrophages were responsible for this cellular influx and that viral clearance still occurs. The second peak of chemokine production was T cell associated; when T cells were depleted during primary RSV infection, mediator levels were also reduced [6]. This second T cell derived wave of chemokine production may potentiate the recruitment of further T cells, and contributing to the disease seen following RSV infection. As has previously been observed in RSV infected CCL3^−/−^ mice [15], CCL3 depletion reduced T cell recruitment.

Whilst CCL3 inhibition reduced cellular recruitment, it also increased weight loss. This is of interest because cellular burden in the lungs is often taken as an indicator of severity of inflammation and of disease. In this case, disease was increased despite reduced cell recruitment. The cause of this increased disease is likely to be increased inflammatory mediator production. Our finding that CCL3 inhibition enhances pathology is similar to the finding that in a model of nephritis CCR1^−/−^ (the main receptor for CCL3) mice have enhanced pathology associated with increased TNF levels [16]. In the current study we observed that CCL3 depletion increased the number of RSV-specific TNF producing CD8 T cells, which may be pathogenic [17], [18]. In support of this explanation, we found that TNF depletion decreased weight loss.

The critical role of CD8 T cells in RSV pathology has previously been demonstrated. When CD8 cells are depleted, weight loss decreases [5], [6]; when virus-specific CD8 cells are adoptively transferred into RSV infected mice, weight loss increases [19]. These data support the idea that disease following RSV infection is dependent upon TNF producing CD8 cells and that the increase in pathology following CCL3 inhibition is caused by an increase in the proportion of these pro-inflammatory cells. Depletion of CD8 cells during CCL3 depletion is required to test this conclusion. However, it is notable that there was a difference between TNF and CCL3 depletion on peak viral load: TNF depletion increased viral load, while CCL3 depletion had no effect. Since neither CCL3 nor TNF increased NK cell recruitment, this effect on viral load may be mediated by activation of macrophages by TNF – supported by our previous data where macrophage depletion increased peak viral load [13], alternatively TNF has recently been demonstrated to have anti-viral activity against RSV via the induction of β-defensin 2 [20].

How CCL3 depletion leads to increases in the number of pro-inflammatory CD8 cells is unclear. One possibility is a change in the number of regulatory T cells, but there was no difference in the level of FoxP3 mRNA suggesting that this was not the case (data not shown). It is becoming increasingly apparent that the cytokine production profile of T cells has a critical effect on outcome. Recently CD8 effector cells that are able to produce IL-10 and thereby limit pathology have been described [8]. It has been shown that CCL3 is important in the recruitment of naïve CD8 cells to the site of CD4 T cell-DC interaction [21]. CCL3 may be important in the differential recruitment of regulatory cells compared to the pro-inflammatory TNF producing CD8 T cells.

We conclude that CCL3 production has critically important effects in RSV disease. Its inhibition enhances the recruitment of pro-inflammatory T cells during later stages of infection, so enhancing disease. The cells recruited by CCL3 are able to reduce the pathogenic immune response. Therefore CCL3 alters the quality and not just the magnitude of the inflammatory response in RSV infection.

## Materials and Methods

### Mice and Virus

Female C57BL/6 and BALB/c mice were obtained from Harlan Olac Ltd (Gwenapp, UK). CCL3^−/−^ mice (Scya3) were obtained from B&K Universal (UK). All mice were bred in specific pathogen free conditions and used when 6 to 10 weeks old. RSV (A2 strain) was grown in HEp-2 cells. All work was performed according to institutional and Home Office guidelines. Isoflurane anaesthetized mice were infected intranasally (i.n.) with 5×10^5^ PFU RSV. For CCL3 depletion, mice were treated with 500 µl polyclonal rabbit anti-CCL3 i.p. on days −1 and +1 of challenge. For TNF depletion, mice were treated with 500 µl monoclonal rat anti-TNF i.p. (IgG1; clone XT22 from Schering-Plough, purified by AbD Serotec) on day 0, 2, 4 and 6 of challenge.

### Cell and Tissue Recovery

After infection, animals were sacrificed by i.p. pentobarbitone injection and tissues collected as described [22]. Bronchoalveolar lavage (BAL) was obtained by inflating the lungs via the trachea with 2% lidocaine in EMEM. Lungs were processed to single cell suspension and live cells counted by trypan blue exclusion. Cytospin slides were prepared from BAL and stained with H&E.

### Detection of Cytokines and Chemokines by ELISA

Cytokine levels were assessed in BAL or lung mash supernatants by ELISA using pairs of capture and biotinylated detection antibodies (Cytokine: BD or Chemokine: R&D systems) following manufacturers' instructions. Concentrations were quantified by comparison to recombinant cytokine standards.

### Flow Cytometry of Mouse Lung Cells

Cell staining was performed as described previously [22]. Cells were Fc blocked using anti-CD16/32. For surface staining antibodies against surface markers (BD) or the RSV M2 MHC class I pentamer (SYIGSINNI; Proimmune) were used. For intracellular staining, cells were stimulated for 4 h at 37°C in the presence of 10 µg/ml Brefeldin A with 1 µg/ml M2_82-90_ peptide (SYIGSINNI) plus 50 U/ml IL-2. Cells were permeabilised with 0.5% saponin and stained with directly conjugated anti-TNF, anti-IFNγ or anti-CCL3-biotin and streptavidin:PE-Cy5 or isotype control. Samples were run on an LSR (BD) and analyzed using Winlist (Verity).

### NK Cell Chemotaxis Assay

Spleen lymphocytes were isolated using Ficoll-plaque separation. NK cell enrichment from the spleen lymphocyte preparations was performed using the MACS manual negative NK selection kit (Miltenyi Biotech Ltd, UK). Chemotactic responses of splenic NK cells were assessed in triplicate using ChemoTx system micro-chemotaxis 96 well plates (5 µm pore size, Neuro Probe Inc, USA). Wells were blocked with RPMI/0.1% BSA at 37°C. The chemotactic agent to be tested was carefully added to the wells and the filter placed on top. Enriched NK suspension was added onto the filter at a concentration of between 5×10^6^ cells/ml and 1×10^7^ cells/ml. Chemotaxis was measured after 1.5 hours incubation at 37°C. Cells were collected and stained with DRAQ5 live/dead marker solution. Cells were stained for DX5 and CD3 prior to quantifying by flow cytometry by comparison to a known quantity of CountBright Absolute counting beads (Molecular Probes, Invitrogen, USA) on a Cyan ADP LX 9 color flow cytometer (DAKO, UK). Chemotactic index calculated as cell number migrated to chemoattractant/cell number to media/PBS alone.

### Detection of CCL3 RNA

Total RNA was extracted from snap-frozen lung using RNA-Stat-60™ according to manufacturer's instructions (Tel-Test, TX). cDNA, from 2 µg RNA, was generated with random hexamers using Omniscript RT (Qiagen). TaqMan PCR was performed using an ABI 7000 (Applied Biosystems) and analyzed using SDS v1.2.3. The following primers and probe were used: CCL3, Forward primer: CATCGTTGACTATTTTGAAACCAG, Reverse primer: GCCGGTTTCTCTTAGTCAGGAA and probe 5′-FAM-AGCCTTTGCTCCCAGCCAGGTGTC-TAMRA-3′. Copy numbers were determined against standard curves.

### RSV Plaque Assay

RSV titer from lungs was assessed using plaque assay as described previously [22].

### Detection of Cytokine Secretion by ELISPOT

ELISPOT were performed as described before [22]. Multiscreen 96-well immobilon-P filtration plates were coated with purified anti-IFNγ (4 µg/ml; R&D) overnight at 4°C. Lung cells were stimulated with 2 PFU/cell RSV or HEp-2 cell lysate for 2 days at 37°C. Detection was performed using biotinylated anti-IFNγ, followed by streptavidin-alkaline phosphatase. Bound antibody was visualized by incubating plates with substrate BCIP/NBT (Sigma). After drying, spots were enumerated on an inverted microscope.

### Statistical Analysis

Statistical significance between experimental groups was determined by ANOVA and post test or Student's t test using GraphPad Prism software.
